# Characterization of Olive Oils Obtained from Minor Accessions in Calabria (Southern Italy)

**DOI:** 10.3390/foods10020305

**Published:** 2021-02-02

**Authors:** Amalia Piscopo, Rocco Mafrica, Alessandra De Bruno, Rosa Romeo, Simone Santacaterina, Marco Poiana

**Affiliations:** Department of AGRARIA, University Mediterranea of Reggio Calabria, 89124 Vito, Reggio Calabria, Italy; amalia.piscopo@unirc.it (A.P.); rocco.mafrica@unirc.it (R.M.); alessandra.debruno@unirc.it (A.D.B.); rosa.romeo@unirc.it (R.R.); simone.santacaterina@unirc.it (S.S.)

**Keywords:** clones, minor accessions, olive oil, quality

## Abstract

The valorization of minor accessions of olive is potentially a good way to improve the qualitative production of a specific territory. Olive oils of four minor accessions (Ciciarello, Tonda di Filogaso, and Ottobratica Calipa and Ottobratica Cannavà clones) produced in the same area of the Calabria region were characterized for the principal qualitative analyses at two drupe harvesting periods (October and November). Good quality in terms of free acidity, peroxides, spectrophotometric indexes, and fatty acid composition was observed in olive oils produced at both drupe harvesting times, with the exception of those of Tonda di Filogaso, which showed a free acidity level over the legal limit for extra virgin olive oil in the second harvesting time. All of the olive oils possessed at both production periods averagely abundant total polyphenols (460–778 mg/kg) and tocopherols (224–595 mg/kg), and the amounts changed in the experimental years for expected different environmental variations. Ottobratica Cannavà and Ottobratica Calipa clones showed some peculiar qualitative characteristics (free acidity, peroxides, fatty acid composition, and total polyphenols), distancing themselves from the principal variety of reference, Ottobratica.

## 1. Introduction

In view of the recognized importance of the right lifestyle, primarily resulting in healthy eating, the daily consumption of olive oil is highly recommended for its dotation in monounsaturated fatty acids, in particular oleic acid, and antioxidant compounds, proven to reduce the incidence of cardiovascular and age-associated diseases [1]. Olive variety has a remarkable impact on absolute and relative concentrations of oil components, such as fatty acids, triacylglycerols, and sterols [2,3,4], and sensorial characteristics [5] and antioxidant compounds, such as polyphenols, tocopherols [6,7], and squalene [8,9]. Nowadays, studies on minor olive cultivars, also called neglected, have sparked interest in different countries for the topic of biodiversity protection and the possibility to improve, enrich, and diversify local olive oil productions [10,11,12,13].

The Italian olive heritage contains over 500 varieties; many of these are in Calabria [14], a region located in the southern Italy, particularly due to favorable geographic area, climate, and soil conditions that promote the diffusion of cultivars (about 33) to a different extent. Some of these are largely present along the Calabria region, such as Carolea cv. [15], some others grow in more specific areas, such as Grossa di Gerace, Ottobratica, and Sinopolese cv. [16,17], and others grow in limited towns, such as Roggianella [18]. In previous works, it was evidenced that the cultivation in the different areas of Calabria, where different microclimates are present, significantly impacts the diversification and typical characterization of productions, both from different varieties [16] and from the same cultivar [19]. Correlated with these results, the authors have conducted with this study a first investigation on qualitative parameters of olive oils obtained from four minor olive accessions, Ciciarello, Tonda di Filogaso, Ottobratica Calipa, and Ottobratica Cannavà, that are grown in the same area of Calabria.

This paper aims to investigate for the first time the chemical characteristics of olive oils from four minor olive accessions, Ciciarello, Tonda di Filogaso, Ottobratica Calipa, and Ottobratica Cannavà, present in the Tyrrenian Southern area of Calabria. Ottobratica Calipa and Ottobratica Cannavà are in particular two genotypes selected within the Ottobratica population variety in the last decades by the olive growers of this specific territory of the Calabria region [20]. The study focused on olive trees cultivated in the same area of Calabria. This approach was considered to exclude possible different effects of climatic conditions among the varieties, except those linked to the annual trend that occurred similarly for all four varieties. This research represents an interesting opportunity for olive oil production in Calabria. Despite their low diffusion in the whole region as a result of past selections, the minor olive accessions must be studied because, being autochthonous, they possess various characteristics of rusticity and adaptability to the microclimate. This study can also contribute to the protection of olive biodiversity in the Calabria region and its valorization at the same time. Moreover, the chemical characterization of obtained olive oils gives new knowledge, and can be considered as a valid instrument to improve and strengthen qualitative olive productions in Calabria.

## 2. Materials and Methods

### 2.1. Sampling

The studied olive oils were obtained from four olive accessions (Ciciarello, Tonda di Filogaso, Ottobratica Calipa, and Ottobratica Cannavà) in a fifteen-year-old olive grove located in Gioia Tauro Plain, an important olive growing area located on the Tyrrhenian side of the Calabria region (southern Italy). Ottobratica cultivar was also submitted to the research as a reference for its related clones. Previous morphological and molecular characterization studies conducted on the two clones of Ottobratica [20] averted the risks of cases of synonymy or homonymy, both between the two clones and with the most widespread type of Ottobratica (used in this study as a reference element). The orchard was characterized by homogeneous trees, in good vegetative and productive condition, trained according to the open-center training system, spaced 6.0 × 6.0 m, and grown under rain-fed conditions. The soil of the olive orchard was deep, without a skeleton, had a medium texture, was non-calcareous, and with a sub-acid reaction. During the three years of trials, 2017, 2018, and 2019, the average annual temperature and rainfall were, respectively, 15 °C and 1427 mm. The fertilization was carried out at the end of winter with the controlled release fertilizer (N:P:K 21:5:9 with microelements) at three kilograms per tree. In order to ensure the health integrity of trees and fruits, continuous monitoring for the main olive parasites was carried out, using pest control treatments when necessary and according to the principles of integrated pest management.

The experiment was carried out considering three blocks of the four accessions, each composed of three olive trees. About 10–12 kg of drupes were sampled from each block in two harvesting times: October (O) and November (N) of 2017, 2018, and 2019.

### 2.2. Analytical Methods

The oil yield (% oil dry weight) was determined in drupes after stone removing by extraction with petroleum ether in a Soxhlet apparatus (Bicasa s.r.l., Bernareggio, MI, Italy). For the olive oil extraction, about 15 kg of drupes were milled with a hammer mill. The obtained paste was mixed at a temperature below 20–25 °C for 30 min and pressed using a hydraulic press (pressure up to 200 bar) in a small olive oil press mill Mini 30 system (Agrimec Valpesana, Firenze, Italy). After centrifugation and filtration through paper, olive oils were then stored in dark glass bottles at room temperature and analyzed for the total free acidity value, peroxide index, and UV light absorption coefficients according to EC regulations [21,22]. Pigments were extracted from the oil samples (5 mL of oil and 5 mL of cyclohexane) following the method reported by Minguez-Mosquera et al. [23], and the total contents of chlorophylls and carotenoids were determined spectrophotometrically (670 nm and 470 nm, respectively). Total tocopherols were evaluated according to Bakre et al. [24]. The oil samples were diluted in isopropanol (1:10) and filtered (0.45 μ pore size). An aliquot of 5 μL of samples was injected in an ultra-high performance liquid chromatography (UHPLC) system (UHPLC PLATINblue, Knauer, Germany), coupled with a fluorescence detector RF-20A/RF-20Axs model (Shimadzu Corporation, Kyoto, Japan) and analyzed (flow rate of 0.4 mL min^−1^) through a mobile phase of methanol/acetonitrile (50:50). The detector was set at a 290 nm excitation wavelength and 330 nm emission wavelength. The identification and quantification were performed by calibration curve, using pure α-tocopherol as the standard and concentrations ranging from 10 to 500 mg kg^−1^. Results were expressed as mg kg^−1^. Determination of total polyphenols was performed following Baiano et al. [25]. Two mL of methanol/water (70:30, *v*/*v*) and 2 mL of hexane were added to 5 g of oil samples and mixed with a vortex (10 min). The hydro-alcoholic phase containing phenols was separated from the oil phase by several centrifugations; 100 μL of phenolic extract were mixed with 100 μL of Folin–Ciocal teau reagent (2N) and, after 4 min, with 800 μL of an aqueous solution of Na_2_CO_3_ (5%). The mixture was heated in a 40 °C water bath for 20 min, and the total phenol content was determined calorimetrically at 750 nm. The total phenolic content was expressed as milligrams of gallic acid equivalents per kilogram of oil. The total antioxidant activity of the olive oils was detected by 2,2′-azino-bis (3-ethylbenzothiazoline-6-sulfonic acid) (ABTS)/Trolox equivalent antioxidant capacity (TEAC), according to Re et al. [26], and 2,2-diphenyl-1-picrylhydrazyl (DPPH), following the opportunely modified method of Brand-Williams et al. [27]. Fatty acid composition was determined as methyl esters (FAME) following the official method [22].

### 2.3. Statistical Data Elaboration

The results of the analyses were elaborated as mean ± standard deviations of three sampling years for two harvesting times. Significant differences (*p* < 0.05) were obtained by one-way analysis of variance (ANOVA) and multivariate analysis. Pearson’s coefficient was used to study the correlation among qualitative parameters of olive oils. SPSS Software (Version 15.0, SPSS Inc., Chicago, IL, USA) was used for statistical elaboration.

## 3. Results

**Figure 1:**
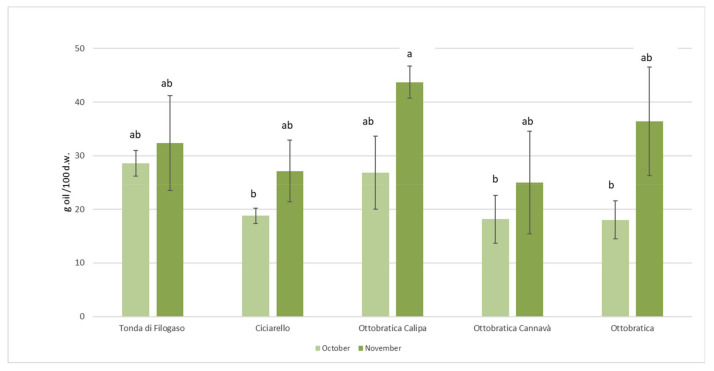
Olive oil yield at two harvesting times of the four studied minor varieties, with Ottobratica cv used as the reference for Calipa and Cannavà clones. Values are the means of 2017, 2018, and 2019. Different letters show significant differences at *p* < 0.05 by Tukey’s post hoc test.

Mean results of the olive oil yield during the three years of study for the minor olive varieties are reported in Figure 1. Drupes of Tonda di Filogaso and Ottobratica Calipa possessed similar oil content at the first sampling (27–29% d.m.), whereas Ciciarello and Ottobratica Cannavà differed for less abundant oiliness (18–19%). In the following harvesting period, the oil content remained significantly similar in Tonda di Filogaso cv, whereas it tended to increase with the highest result in Ottobratica Calipa (44%). The ripening index varied among varieties and harvesting months, as Supplementary Materials shows (Table S1).

The results of principal qualitative parameters of oils, as three-year means, are illustrated in Table 1 and Table 2.

During the three years and at the second production in particular, that is November, a large variability in free acidity was observed in olive oils from the same accession, except in the Ottobratica Cannavà clone (0.55 ± 0.13). The range observed in oils produced in October was 0.31–0.57%; at November, it tended to increase in all of the samples, exceeding the 0.8% in some years, except for Ottobratica Cannavà oils. The other productions were affected probably by a different varietal response to some negative environmental factors linked to a specific year (Tables S2–S6): 2018 for Ottobratica Calipa and Ciciarello (total acidity >1%), and 2019 for Tonda di Filogaso (total acidity near 2.5%), as evidenced by the values of the standard deviations. The observed low quality of Ottobratica olive oil produced in November (Table 2) was confirmed by previous works [28,29], and reflected the origin of its name, strictly linked to its optimal ripening in the month of October. It is interesting to note that for one of the two Ottobratica-related clones, Ottobratica Cannavà, the free acidity inside the legal limit of 0.8% in that period expressed a positive result of the performed new genetic duplication.

Peroxide values of oils were in the range of 2.50–5.58 mEq O_2_/kg, with significant differences between the two harvesting times only in Ottobratica Cannavà oils. Higher peroxide values were noted in the oils of clones compared to those from Ottobratica cv in October, whereas an opposite result was detected for the productions of November (6.63 mEq O_2_/kg in oils from Ottobratica). Spectrophotometric indices denoted olive oil productions of good quality at both harvesting times without significant differences, with the only exception of Tonda di Filogaso olive oils.

The major fatty acid in olive oil is oleic acid; in our study, its content varied with significance from 68.15% (Tonda di Filogaso oils in November) to 75.79% (Ciciarello oils in October). The followed principal detected fatty acids were palmitic acid (C16:0), quantified from 13.23% to 15.74%, and then linoleic acid (C18:2) that varied from 5.96 to 10.06%; both components were similar among minor varieties. The stearic acid (C18:0) was significantly higher in the oils of Tonda di Filogaso obtained in November (3.44%) than in the other samples. The other fatty acids that significantly varied among the samples were C16:1, among the unsaturated ones, from 0.99 to 1.64% and C20:0, among the saturated ones, from 0.34 to 0.46%, as evidenced by the Tukey’s post hoc test (*p* < 0.05) elaboration (data not shown). Olive oils from Ciciarello showed the highest oleic/linoleic (11–13) and monounsaturated/polyunsaturated acid (MUFA/PUFA) (10–12) ratios, confirming the previously discussed results for fatty acid quantification. The antioxidant compositions of olive oil from minor accessions is reported in Table 3.

Significant (*p* < 0.01) differences of pigment amounts were observed among the samples; the olive oils of Ciciarello and Tonda di Filogaso obtained in October were the richest in chlorophylls (10.95 ± 3.41 mg/kg and 10.21 ± 5.05 mg/kg, respectively). Ciciarello olive oils were even the richest in total carotenes (7.16 ± 2.28 mg/kg). The oils extracted in November showed reduced pigment amounts and, in particular, a major reduction was observed in Ottobratica Calipa olive oils (TCL: 2.53 ± 1.23 mg/kg and TCA: 2.26 ± 0.90 mg/kg). ANOVA data elaboration showed variations for pigments between harvesting times, except in the oils of the Ottobratica Cannavà clone (Table 3).

Chlorophyll and carotenoid amounts were also significantly (*p* < 0.05) higher than those resulted in the cultivar population of reference (Ottobratica cv).

Comparing the total mean amounts of polyphenols quantified at two harvesting times, no significant differences were noted. 

Among productions in November, Ottobratica Cannavà oils possessed higher mean phenolic antioxidant amount than the other clone, Ottobratica Calipa, and Ottobratica cv. The total tocopherols detected in the oils from minor accessions were in the range of 225–595 mg/kg; Ottobratica Cannavà olive oils were the richest for this typology of antioxidants (with the only observed significant variation between harvesting times), followed by Ottobratica Calipa olive oils, whereas lower amounts were detected in those from Tonda di Filogaso (224–227 mg/kg) as confirmed by literature [30]. A significant decrease in TT content was observed only in oils from Ottobratica Cannavà extracted in November. In the other productions, the total tocopherols remained constant.

The antioxidant activity of the oils was analyzed by the reaction against two radicals, DPPH and ABTS. The obtained results denoted a higher response with the second antioxidant assay (23.29–45.67%) than the DPPH radical (13.80–34.71%). The largest differences among the varieties were significantly observed in the oils produced in October for ABTS assays (Ottobratica Cannavà > Ottobratica Calipa > Ciciarello = Tonda di Filogaso).

## 4. Discussion

The olive oil accumulation on fruits during ripening follows the triglyceride-forming biosynthesis pathway up to the achievement of full drupe maturation. The olive oil yield in fruits is influenced by the choice of the right harvesting time for each variety and by several growing conditions, such as water availability [31]. In our study, an evident effect of varietal characteristics was observed, and the two Ottobratica clones differed for mean oil production at both harvesting times. Free acidity is generally the first parameter discussed to evaluate the quality of olive oil production; it is well-known that oil-free acidity can be affected by many factors, including fruit handling harvesting mean, storage, and processing, but also by the harvesting time. All of the oils produced in October were inside the limits for the extra virgin olive category [21], and, in particular, those of Ciciarello possessed the lowest mean acidity among the other minor accessions (*p* < 0.05). It is interesting to note that good oils from Ottobratica clones could be obtained at both harvesting times, without significant variations among each other (*p* > 0.05) and, different from Ottobratica, their reference variety. The oils of this last production in November denoted the previously discussed variability during the years and, on average, poor quality (free acidity of 1.18 ± 1.08%). Peroxides and extinction coefficients complied with the regulation limits for the extra virgin olive oil [21]. Fatty acid composition of samples was inside the limits imposed by European regulation for the extra virgin category [32]. The fatty acid composition did not largely vary between harvesting times in oils from each minor accession; only the oils of Tonda di Filogaso were different for stearic acid (means of 1.72% in October and 3.44% in November). Oleic acid was particularly abundant in oils from Ciciarello and Ottobratica Cannavà; for this chemical parameter, oils becoming to this clone were of higher quality with respect to those from Ottobratica cv.

The molecules responsible for olive oil color are pigments belonging to chlorophyll and carotenoid compounds. Their quantification is important to determine not only the sensorial characters and consumer acceptability of olive oils, but also their antioxidant potentiality; olive oil chlorophylls react as radical scavengers in dark storage and as pro-oxidant (sensitizer pigments) in light. Carotenes instead protect cells against the light, with oxygen and sensitizer pigment effects having the ability to quench singlet oxygen and excited sensitizer molecules. Moreover, they can also react as antioxidants under conditions other than photosensitization [33]. The total pigment content varies among varieties, drupe ripening, or olive oil stocking before the extraction [34,35].

In particular, the total content can range from 2 to 40 mg/kg for chlorophylls and from zero to a few mg/kg for carotenoids [36,37]. Olive oil produced at the second harvesting time showed reduced amounts of both pigments in our study, according to the literature [38]. Among the oil samples from the different accessions, only those of Cannavà did not vary for total pigments. Total tocopherols were detected in oils from minor varieties at a higher content than those observed in oils obtained from other cultivars in Calabria [15]. The quantified total phenols were in the range of 460–778 mg/kg, according to Fabiani [39], manifesting a strong antioxidant potentiality. A positive correlation between the total phenol content and ABTS assay was indeed evidenced by a high Pearson coefficient (r = 0.7–0.9) in all of the oils, in particular those extracted in November, according to Sicari [40]. It confirms their usefulness in providing the minimum intake of 5 mg of hydroxytyrosol per serving of olive oil (total phenol content >250 mg/kg) that is required to manifest the antioxidant effect in a balanced diet.

Finally, a multivariate data analysis was performed to evidence the influence of varietal characteristics or drupe harvesting times to the olive oil quality (Table 4). Results showed an evident effect due to the olive origin, especially for the prevalent fatty acids (*p* < 0.00) in the olive oils. From this data elaboration that considered overall data for all of the three years of experimentation, it was noted that, among the other qualitative parameters, in particular the total carotene content was affected exclusively by the drupe ripening, as the literature [35] confirms.

## 5. Conclusions

This study allowed the characterization of the oil productions from four minor olive accessions grown in the same area of Calabria, with the aim to compare the qualitative differences measured during three years of observations, excluding climatic variables due to different environmental conditions. For some of these (oils from Tonda di Filogaso and Ciciarello cv), harvesting times significantly affected the results for free acidity and total pigments. All of the oil productions obtained in October possessed the chemical parameters to be classified as extra virgin olive oils. Comparing all of the studied accessions, olive oils from Ciciarello cv and Calipa and Cannavà clones also showed good quality when extracted in November. This is interesting for growing practices in the same studied area and for new knowledge about the potentiality of the two new clones obtained from the Ottobratica cultivar. In particular, the oils of the Ottobratica Cannavà clone showed better quality for free fatty acidity at both harvesting times, with oleic acid content and total antioxidants (polyphenols and tocopherols) with respect to the cultivar of reference, Ottobratica, largely diffused in the considered territory.

## Figures and Tables

**Table 1:** Principal chemical parameters of olive oils of Tonda di Filogaso (TF), Ciciarello (C), Ottobratica Calipa. (O. CLP) Ottobratica Cannavà (O. CNV) and Ottobratica (O) accessions.

Qualitative Parameters	Accessions	Harvesting Times		
		O	N	Sign.
FA (oleic acid %)	TF	0.55 ± 0.18a	1.47 ± 1.11	*
	C	0.31 ± 0.06b	0.69 ± 0.39	*
	O. CLP	0.57 ± 0.24a	0.74 ± 0.54	n.s.
	O. CNV	0.53 ± 0.13ab	0.55 ± 0.13	n.s
	O	0.40 ± 0.09ab	1.18 ± 1.08	*
Sign.		*	n.s.	
PV (mEq O_2_/kg)	TF	3.57 ± 1.05ab	3.59 ± 1.94	n.s.
	C	2.50 ± 0.52b	3.13 ± 1.53	n.s.
	O. CLP	2.71 ± 0.99ab	3.14 ± 1.70	n.s
	O. CNV	3.88 ± 1.27a	5.58 ± 1.12	*
	O	2.30 ± 0.64b	6.63 ± 3.88	**
Sign.		**	n.s.	
K_232_	TF	1.94 ± 0.15	1.62 ± 0.22	**
	C	1.58 ± 0.55	1.51 ± 0.20	n.s
	O. CLP	2.08 ± 0.39	1.82 ± 0.25	n.s
	O. CNV	1.82 ± 0.47	1.74 ± 0.40	n.s
	O	1.81 ± 0.26	1.76 ± 0.51	n.s.
Sign.		n.s.	n.s.	
K_270_	TF	0.22 ± 0.04	0.15 ± 0.05	*
	C	0.19 ± 0.07	0.19 ± 0.03	n.s
	O. CLP	0.23 ± 0.09	0.22 ± 0.08	n.s
	O. CNV	0.18 ± 0.14	0.13 ± 0.09	n.s
	O	0.19 ± 0.06	0.19 ± 0.07	n.s.
Sign.		n.s.	n.s.	
ΔK	TF	0.00 ± 0.00	0.00 ± 0.00	n.s.
	C	0.00 ± 0.00	0.00 ± 0.00	n.s
	O. CLP	0.00 ± 0.00	0.00 ± 0.00	n.s
	O. CNV	0.00 ± 0.00	0.00 ± 0.00	n.s
	O	0.00 ± 0.00	0.00 ± 0.00	n.s
Sign.		n.s.	n.s.	

The data are presented as means ± standard deviations. ** Significance at *p* < 0.01; * significance at *p* < 0.05; n.s., not significant; a, ab, b see Figure 1.

**Table 2:** Fatty acid compositions of olive oils of Tonda di Filogaso (TF), Ciciarello (C), Ottobratica Calipa. (O. CLP) Ottobratica Cannavà (O. CNV) and Ottobratica (O) accessions.

	Accessions	Harvesting Times				Accessions	Harvesting Times		
		O	N	Sign.			O	N	Sign.
C16:0 (%)	TF	15.07 ± 1.87	15.11 ± 0.13	n.s.	C18:2 (%)	TF	8.55 ± 1.62ab	10.06 ± 1.59	n.s.
	C	13.23 ± 0.84	13.51 ± 0.93	n.s.		C	5.96 ± 1.78b	7.59 ± 2.90	n.s.
	O. CLP	13.86 ± 3.04	14.49 ± 1.42	n.s.		O. CLP	8.93 ± 3.35ab	8.92 ± 2.35	n.s.
	O. CNV	13.40 ± 2.37	13.42 ± 2.22	n.s.		O. CNV	7.78 ± 2.59ab	7.52 ± 2.31	n.s.
	O	15.74 ± 0.69	14.96 ± 1.21	n.s.		O	9.28 ± 1.19a	9.14 ± 1.95	n.s.
Sign.		n.s.	n.s.		Sign.		*	n.s..	
C16:1 (%)	TF	1.50 ± 0.23	1.63 ± 0.24	n.s.	C18:3 (%)	TF	0.83 ± 0.38	0.56 ± 0.09	n.s.
	C	0.99 ± 0.25	1.03 ± 0.19	n.s.		C	0.58 ± 0.05	0.52 ± 0.06	*
	O. CLP	1.63 ± 0.81	1.64 ± 0.43	n.s.		O. CLP	0.74 ± 0.11	0.64 ± 0.17	n.s.
	O. CNV	1.07 ± 0.33	1.40 ± 0.67	n.s.		O. CNV	0.74 ± 0.50	0.61 ± 0.17	n.s.
	O	1.59 ± 0.27	1.49 ± 0.41	n.s.		O	0.62 ± 0.08	0.63 ± 0.08	n.s.
Sign.		*	n.s.		Sign.		n.s..	n.s..	
C17:0 (%)	TF	0.09 ± 0.07ab	0.09 ± 0.06	n.s.	C20:0 (%)	TF	0.34 ± 0.12b	0.38 ± 0.04b	n.s.
	C	0.06 ± 0.04b	0.08 ± 0.05	n.s.		C	0.46 ± 0.05a	0.46 ± 0.02a	n.s.
	O. CLP	0.04 ± 0.02b	0.04 ± 0.03	n.s.		O. CLP	0.41 ± 0.10ab	0.37 ± 0.04b	n.s.
	O. CNV	0.09 ± 0.11ab	0.05 ± 0.01	n.s.		O. CNV	0.45 ± 0.02ab	0.41 ± 0.05ab	n.s.
	O	0.18 ± 0.14a	0.10 ± 0.07	n.s.		O	0.41 ± 0.05ab	0.42 ± 0.06ab	n.s.
Sign.		*	n.s.		Sign.		*	**	
C17:1 (%)	TF	0.17 ± 0.14	0.19 ± 0.11	n.s.	C20:1 (%)	TF	0.28 ± 0.06	0.23 ± 0.01b	n.s.
	C	0.10 ± 0.08	0.15 ± 0.08	n.s.		C	0.30 ± 0.03	0.29 ± 0.05a	n.s.
	O. CLP	0.09 ± 0.04	0.10 ± 0.05	n.s.		O. CLP	0.27 ± 0.09	0.30 ± 0.05a	n.s.
	O. CNV	0.13 ± 0.09	0.10 ± 0.03	n.s.		O. CNV	0.29 ± 0.05	0.28 ± 0.03ab	n.s.
	O	0.72 ± 1.43	0.19 ± 0.11	n.s.		O	0.26 ± 0.04	0.28 ± 0.02ab	n.s.
Sign.		n.s.	n.s.		Sign.		n.s.	*	
C18:0 (%)	TF	1.72 ± 0.76	3.44 ± 0.34	**	C22:0 (%)	TF	0.22 ± 0.28	0.12 ± 0.01ab	n.s.
	C	2.32 ± 1.11	2.56 ± 1.82	n.s.		C	0.15 ± 0.02	0.15 ± 0.02a	n.s.
	O. CLP	1.33 ± 0.63	2.06 ± 1.19	n.s.		O. CLP	0.12 ± 0.05	0.12 ± 0.02b	n.s.
	O. CNV	1.95 ± 0.72	1.84 ± 1.16	n.s.		O. CNV	0.15 ± 0.01	0.13 ± 0.02ab	n.s.
	O	2.49 ± 0.93	1.77 ± 0.61	n.s.		O	0.15 ± 0.01	0.14 ± 0.03ab	n.s.
Sign.		n.s.	n.s.		Sign.		n.s.	*	
C18:1 (%)	TF	71.11 ± 3.99ab	68.15 ± 1.25b	n.s.	MUFA/PUFA	TF	8.13 ± 1.78	6.77 ± 1.14	n.s.
	C	75.79 ± 2.82a	73.61 ± 4.36ab	n.s.		C	12.56 ± 2.84	10.47 ± 3.41	n.s.
	O. CLP	72.46 ± 4.48ab	71.26 ± 3.92ab	n.s.		O. CLP	8.78 ± 3.72	8.31 ± 2.82	n.s.
	O. CNV	73.87 ± 4.88a	74.18 ± 4.22a	n.s.		O. CNV	9.95 ± 3.88	10.24 ± 3.57	n.s.
	O	68.50 ± 1.26b	70.83 ± 3.01ab	*		O	7.29 ± 1.09	7.88 ± 2.50	n.s.
Sign.		**	*		Sign.		n.s.	n.s.	

The data are presented as means ± standard deviations. **, *, n.s. see Table 1; a, ab, b see Figure 1.

**Table 3:** Antioxidant composition and activity of Tonda di Filogaso (TF), Ciciarello (C), Ottobratica Calipa. (O. CLP) Ottobratica Cannavà (O. CNV) and Ottobratica (O) accessions.

		Accessions	Harvesting Times		
			O	N	Sign.
Antioxidant property	TChl	TF	10.21 ± 5.05	3.03 ± 0.90b	**
		C	10.95 ± 3.41	3.41 ± 1.02b	**
		O. CLP	7.00 ± 2.18	2.53 ± 1.23b	**
		O. CNV	6.35 ± 3.58	6.19 ± 3.84a	n.s
		O	8.76 ± 3.72	3.52 ± 1.17ab	**
	Sign.		n.s.	**	
	TCa	TF	5.97 ± 1.82	2.71 ± 0.64b	**
		C	7.16 ± 2.28	3.25 ± 1.15ab	**
		O. CLP	5.17 ± 0.92	2.26 ± 0.90b	**
		O. CNV	4.98 ± 2.69	5.46 ± 3.58a	n.s
		O	5.72 ± 1.58	3.32 ± 1.02ab	**
	Sign.		n.s.	*	
	TT	TF	227 ± 19c	224 ± 10c	n.s
		C	289 ± 18bc	242 ± 30bc	n.s
		O. CLP	324 ± 19b	309 ± 36a	n.s
		O. CNV	595 ± 66a	286 ± 32ab	**
		O	266 ± 44bc	238 ± 33bc	n.s.
	Sign.		**	**	
	TP	TF	615 ± 403	516 ± 130	n.s.
		C	460 ± 123	486 ± 196	n.s.
		O. CLP	617 ± 397	446 ± 279	n.s
		O. CNV	778 ± 235	695 ± 318	n.s
		O	560 ± 453	334 ± 245	n.s.
	Sign.		n.s.	n.s.	
Antioxidant activity	DPPH assay	TF	20.58 ± 13.62	14.09 ± 6.65b	n.s
		C	25.40 ± 13.70	13.80 ± 3.11b	n.s
		O. CLP	21.17 ± 11.50	20.50 ± 7.77ab	n.s
		O. CNV	34.71 ± 3.99	26.07 ± 2.70a	n.s
		O	21.97 ± 6.76	15.62 ± 12.33ab	n.s.
	Sign.		n.s.	*	
	ABTS assay	TF	29.01 ± 3.67b	31.72 ± 12.11	n.s
		C	31.69 ± 10.26ab	33.79 ± 17.80	n.s
		O. CLP	23.29 ± 10.58b	24.76 ± 10.07	n.s
		O. CNV	45.67 ± 15.69a	37.52 ± 13.27	*
		O	25.49 ± 7.44b	32.07 ± 23.36	n.s.
	Sign.		**	n.s.	

Amount expressed as mg/kg for TChl (total chlorophylls), TCa (total carotenoids), TT (total tocopherols), TP (total polyphenols), and % inhibition/mg for DPPH and ABTS assays. The data are presented as means ± standard deviations; **, *, n.s. see Table 1; a, ab, b, c see Figure 1.

**Table 4:** Multivariate analyses of qualitative characteristics of olive oils extracted from drupes produced from the four minor accessions and at two harvesting times.

Variables	Accession	Harvesting Time
FA	**	**
PV	**	**
ΔK	**	n.s.
TChl	*	**
TCa	n.s.	**
TT	**	**
TP	*	**
C16:0	**	n.s.
C18:1	**	n.s.
C18:2	**	n.s.

** Significance at *p* < 0.01; * significance at *p* < 0.05; n.s., not significant.

## Data Availability

The datasets presented in this study are available as Supplementary materials.

## Supplementary Materials

The following are available online at https://www.mdpi.com/2304-8158/10/2/305/s1, Table S1: Ripening index of the olives at harvest times, Table S2: Qualitative characterization of the Tonda di Filogaso oils, Table S3: Qualitative characterization of the Ciciarello oils, Table S4: Qualitative characterization of the Ottobratica Calipa oils, Table S5: Qualitative characterization of the Ottobratica Cannavà oils, Table S6: Qualitative characterization of the Ottobratica oils.
