# Supplementary material for: Characterization of Olive Oils Obtained from Minor Accessions in Calabria (Southern Italy)
Source: Foods. 2021 Feb 2;10(2):305. doi: 10.3390/foods10020305 (PMC7912949; doi:10.3390/foods10020305)
Supplement: Supplementary file 1 [file foods-10-00305-s001.pdf]

foods MDPI

Table 1. Ripening index of the olives at harvest times.

		2017	2018	2019
**Ciciarello**	**October**	2,33	3,53	1,50
	**November**	4,48	4,75	3,52
**Tonda di Filogaso**	**October**	3,91	4,33	4,20
	**November**	4,92	6,30	6,10
**Ottobratica Calipa**	**October**	2,94	3,01	2,83
	**November**	4,65	4,15	4,53
**Ottobratica Cannavà**	**October**	3,50	3,47	3,48
	**November**	5,03	5,00	5,02
**Ottobratica standard**	**October**	2,95	3,70	3,13
	**November**	4,40	5,27	4,94

Table 2. Qualitative characterization of the Tonda di Filogaso oils.

	2017		2018		2019	
**TONDA DI FILOGASO**	**O**	**N**	**O**	**N**	**O**	**N**
**FA (oleic acid %)**	0,69 ± 0,22	0,44 ± 0,04	0,39 ± 0.02	1,47 ± 1.11	0,57 ± 0.08	2,50 ± 0.58
**PV (mEq O_2/kg)**	4,72 ± 0,29	1,68 ± 0,29	2,22 ± 0.11	3,59 ± 1.94	3,78 ± 0.13	5,49 ± 0.46
**K_{232}**	1,92 ± 0.07	1,40 ± 0.06	1,90 ± 0.23	1,62 ± 0.22	2,00 ± 0.09	1,83 ± 0.05
**K_{270}**	0,20 ± 0.01	0,10 ± 0.01	0,26 ± 0.02	0,15 ± 0.05	0,19 ± 0.00	0,19 ± 0.01
**ΔK**	-0,00 ± 0.00	-0,00 ± 0.00	0,00 ± 0.00	0,00 ± 0.00	0,00 ± 0.00	0,00 ± 0.00
**C16:0**	14,36 ± 0.13	15,10 ± 0.12	13,26 ± 0.06	15,11 ± 0.13	17,58 ± 0.60	15,12 ± 0.14
**C16:1**	1,43 ± 0.05	1,39 ± 0.02	1,42 ± 0.32	1,63 ± 0.24	1,63 ± 0.16	1,87 ± 0.05
**C17:0**	0,03 ± 0.00	0,16 ± 0.01	0,04 ± 0.00	0,09 ± 0.06	0,19 ± 0.02	0,03 ± 0.00
**C17:1**	0,07 ± 0.01	0,30 ± 0.01	0,09 ± 0.01	0,19 ± 0.11	0,35 ± 0.06	0,08 ± 0.00
**C18:0**	1,61 ± 0.46	3,14 ± 0.09	1,16 ± 0.08	3,44 ± 0.34	2,40 ± 0.87	3,74 ± 0.21
**C18:1**	73,56 ± 0.85	67,11 ± 0.62	73,71 ± 2.32	68,15 ± 1.25	66,07 ± 1.89	69,20 ± 0.75
**C18:2**	7,41 ± 0.3.	11,52 ± 0.41	8,52 ± 2.12	10,06 ± 1.59	9,73 ± 0.77	8,60 ± 0.79
**C18:3**	0,68 ± 0.01	0,47 ± 0.01	1,05 ± 0.54	0,56 ± 0.09	0,76 ± 0.25	0,64 ± 0.00
**C20:0**	0,39 ± 0.00	0,41 ± 0.01	0,32 ± 0.04	0,38 ± 0.04	0,31 ± 0.20	0,34 ± 0.01
**C20:1**	0,26 ± 0.01	0,22 ± 0.01	0,26 ± 0.06	0,23 ± 0.01	0,33 ± 0.08	0,24 ± 0.01
**C22:0**	0,13 ± 0.01	0,13 ± 0.00	0,10 ± 0.02	0,12 ± 0.01	0,43 ± 0.40	0,11 ± 0.00
**C24:0**	0,06 ± 0.00	0,06 ± 0.00	0,08 ± 0.02	0,04 ± 0.02	0,20 ± 0.26	0,02 ± 0.00
**TChl (mg/kg)**	5,63 ± 0.98	2,41 ± 0.47	16,98 ± 1.96	3,03 ± 0.90	8,03 ± 0.33	3,64 ± 0.81
**TCa (mg/kg)**	3,86 ± 0.85	2,16 ± 0.20	7,94 ± 0.89	2,71 ± 0.64	6,13 ± 0.23	3,27 ± 0.38
**TP (mg/kg)**	1.111 ± 107	619 ± 72	142,36 ± 4.88	515,64 ± 129.88	592,81 ± 79.04	412,2 ± 84.99
**TT (mg/kg)**	227,11 ± 19.00	224.39 ± 10.29	219,27 ± 19.01	224,39 ± 10.29	234,96 ± 15.40	224.39 ± 10.29
**DPPH assay (% inhibition/mg)**	34,72 ± 2.83	19.47 ± 3.85	4.37 ± 0.77	19.64 ± 2.63	22.64 ± 4.34	14.15 ± 7.24
**ABTS assay (% inhibition/mg)**	31,58 ± 0.30	43,63 ± 3.07	24,55 ± 1.34	31,72 ± 12.11	30,92 ± 2.88	19,81 ± 0.28

Table 3. Qualitative characterization of the Ciciarello oils.

	2017		2018		2019	
**CICIARELLO**	**O**	**N**	**O**	**N**	**O**	**N**

**FA (oleic acid %)**	0,69 ± 0,22	0,44 ± 0,04	0,23 ± 0.01	1,24 ± 0.02	0,38 ± 0.03	0,40 ± 0.04
**PV (mEq O_2/kg)**	4,72 ± 0,29	1,68 ± 0,29	2,46 ± 0.17	5,13 ± 0.12	2,95 ± 0.25	2,78 ± 0.34
**K_{232}**	1,92 ± 0.07	1,40 ± 0.06	1,18 ± 0.62	1,34 ± 0.15	2,08 ± 0.18	1,72 ± 0.14
**K_{270}**	0,20 ± 0.01	0,10 ± 0.01	0,13 ± 0.08	0,19 ± 0.02	0,25 ± 0.01	0,20 ± 0.05
**ΔK**	0,69 ± 0,22	0,44 ± 0,04	-0,01 ± 0.00	0,00 ± 0.00	0,00 ± 0.00	0,00 ± 0.00
**C16:0**	13,17 ± 0.22	14,67 ± 0.25	13,67 ± 1.24	12,64 ± 0.02	12,86 ± 0.48	13,22 ± 0.59
**C16:1**	1,15 ± 0.23	1,30 ± 0.04	1,04 ± 0.25	0,91 ± 0.00	0,79 ± 0.00	0,88 ± 0.03
**C17:0**	0,04 ± 0.00	0,15 ± 0.02	0,08 ± 0.06	0,06 ± 0.00	0,05 ± 0.00	0,04 ± 0.01
**C17:1**	0,07 ± 0.01	0,26 ± 0.02	0,17 ± 0.12	0,11 ± 0.00	0,07 ± 0.00	0,08 ± 0.00
**C18:0**	2,22 ± 0.22	2,77 ± 0.20	1,39 ± 0.39	0,99 ± 0.08	3,33 ± 1.26	3,90 ± 2.35
**C18:1**	75,04 ± 2.09	67,92 ± 0.54	75,79 ± 3.89	77,65 ± 0.10	76,55 ± 1.78	75,27 ± 2.30
**C18:2**	6,92 ± 1.90	11,63 ± 0.48	6,23 ± 1.83	5,99 ± 0.01	4,74 ± 0.10	5,13 ± 0.28
**C18:3**	0,52 ± 0.01	0,46 ± 0.01	0,62 ± 0.03	0,60 ± 0.01	0,60 ± 0.02	0,50 ± 0.01
**C20:0**	0,42 ± 0.05	0,44 ± 0.00	0,47 ± 0.01	0,47 ± 0.00	0,51 ± 0.01	0,48 ± 0.01
**C20:1**	0,27 ± 0.04	0,22 ± 0.00	0,32 ± 0.01	0,34 ± 0.00	0,32 ± 0.01	0,32 ± 0.02
**C22:0**	0,13 ± 0.02	0,13 ± 0.01	0,15 ± 0.00	0,18 ± 0.00	0,17 ± 0.01	0,16 ± 0.01
**C24:0**	0,05 ± 0.01	0,05 ± 0.00	0,08 ± 0.00	0,08 ± 0.00	0,01 ± 0.01	0,01 ± 0.00
**TChl (mg/kg)**	5,63 ± 0.98	2,41 ± 0.47	13,91 ± 1.95	2,96 ± 0.38	11,83 ± 1.74	4,57 ± 0.67
**TCa (mg/kg)**	3,86 ± 0.85	2,16 ± 0.20	6,72 ± 1.00	2,58 ± 0.19	9,70 ± 1.45	4,75 ± 0.62
**TP (mg/kg)**	1.111 ± 107	619 ± 72	367,50 ± 11.45	300,45 ± 7.67	392,75 ± 46.25	419,44 ± 37.29
**TT (mg/kg)**	288.60 ± 18.30	241.77 ± 30.39	272.88 ± 7.46	261.00 ± 33.24	304,32 ± 10.92	222.54 ± 1.85
**DPPH assay (% inhibition/mg)**	14.62 ± 2.22	13.42 ± 2.53	43.38 ± 0.82	14.13 ± 0.36	18.20 ± 1.82	13.83 ± 4.35
**ABTS assay (% inhibition/mg)**	31,58 ± 0.30	43,63 ± 3.07	45,00 ± 1.33	23,04 ± 0.98	29,09 ± 3.76	19,65 ± 2.07

Table 4. Qualitative characterization of the Ottobratica Calipa oils.

	**2017**		**2018**		**2019**	
**OTTOBRATICA CALIPA**	**O**	**N**	**O**	**N**	**O**	**N**
**FA (oleic acid %)**	0,36 ± 0.11	0,40 ± 0.07	0,86 ± 0.02	1,44 ± 0.19	0,49 ± 0.10	0,37 ± 0.02
**PV (mEq O_2/kg)**	1,56 ± 0.55	1,60 ± 0.14	2,96 ± 0.16	2,96 ± 0.14	3,61 ± 0.27	4,87 ± 1.52
**K_{232}**	1,62 ± 0.03	1,58 ± 0.07	2,48 ± 0.03	1,87 ± 0.23	2,14 ± 0.11	2,00 ± 0.10
**K_{270}**	0,15 ± 0.01	0,13 ± 0.03	0,33 ± 0.06	0,29 ± 0.06	0,20 ± 0.02	0,24 ± 0.02
**ΔK**	0,00 ± 0.00	0,00 ± 0.00	0,00 ± 0.00	0,00 ± 0.00	0,01 ± 0.01	0,00 ± 0.00
**C16:0**	14,96 ± 0.01	12,63 ± 0.15	16,00 ± 0.15	15,50 ± 0.23	10,62 ± 2.88	15,35 ± 0.19
**C16:1**	2,00 ± 0.00	1,12 ± 0.04	2,29 ± 0.15	1,98 ± 0.18	0,61 ± 0.30	1,82 ± 0.19
**C17:0**	0,02 ± 0.01	0,02 ± 0.02	0,05 ± 0.03	0,07 ± 0.03	0,05 ± 0.01	0,03 ± 0.00
**C17:1**	0,08 ± 0.00	0,07 ± 0.01	0,14 ± 0.04	0,16 ± 0.04	0,06 ± 0.01	0,08 ± 0.00
**C18:0**	1,96 ± 0.12	2,42 ± 0.09	1,39 ± 0.18	0,61 ± 0.09	0,64 ± 0.36	3,16 ± 0.55
**C18:1**	71,79 ± 0.09	76,40 ± 0.29	67,76 ± 0.33	68,60 ± 1.08	77,84 ± 1.39	68,77 ± 0.13
**C18:2**	7,73 ± 0.09	6,07 ± 0.23	10,73 ± 0.26	11,33 ± 0.65	8,33 ± 4.98	9,37 ± 0.32
**C18:3**	0,68 ± 0.00	0,44 ± 0.02	0,79 ± 0.02	0,81 ± 0.07	0,74 ± 0.17	0,66 ± 0.02
**C20:0**	0,35 ± 0.00	0,41 ± 0.01	0,35 ± 0.02	0,37 ± 0.02	0,53 ± 0.04	0,34 ± 0.01
**C20:1**	0,26 ± 0.00	0,25 ± 0.02	0,32 ± 0.01	0,36 ± 0.01	0,22 ± 0.12	0,30 ± 0.01
**C22:0**	0,10 ± 0.00	0,12 ± 0.01	0,13 ± 0.01	0,14 ± 0.01	0,12 ± 0.08	0,10 ± 0.00
**C24:0**	0,05 ± 0.00	0,05 ± 0.01	0,07 ± 0.01	0,07 ± 0.01	0,04 ± 0.02	0,01 ± 0.00
**TChl (mg/kg)**	6,55 ± 1.97	2,40 ± 0.44	8,90 ± 1.66	1,24 ± 0.16	5,54 ± 0.25	3,95 ± 0.32
**TCa (mg/kg)**	5,27 ± 0.99	2,22 ± 0.39	5,34 ± 1.06	1,30 ± 0.10	4,90 ± 0.26	3,26 ± 0.30
**TP (mg/kg)**	227,72 ± 53.60	796,01 ± 122.20	521,57 ± 20.83	214,48 ± 9.07	1.104,49 ± 143.13	326,92 ± 52.55

**TT (mg/kg)**	323.71 ± 17.81	308.80 ± 32.68	321,20 ± 18.89	333,77 ± 15.08	326,22 ± 16.29	283,84 ± 25.73
**DPPH assay (% inhibition/mg)**	9,19 ± 0.84	29,98 ± 2.54	35,16 ± 0.82	13,42 ± 0.63	19,16 ± 2.76	18,10 ± 2.90
**ABTS assay (% inhibition/mg)**	13,67 ± 1.84	36,83 ± 2.03	36,42 ± 2.39	23,43 ± 1.39	19,77 ± 3.45	14,02 ± 1.26

Table 5. Qualitative characterization of the Ottobratica Cannavà oils.

	**2017**		**2018**		**2019**	
**OTTOBRATICA CANNAVA′**	**O**	**N**	**O**	**N**	**O**	**N**
**FA (oleic acid %)**	0,41 ± 0.00	0,44 ± 0.04	0,64 ± 0.02	0,66 ± 0.01	0,53 ± 0.13	0,55 ± 0.13
**PV (mEq O_2/kg)**	4,86 ± 0.86	4,58 ± 0.29	2,90 ± 0.08	6,57 ± 0.10	3,88 ± 1.27	5,58 ± 1.12
**K_{232}**	1,39 ± 0.03	1,38 ± 0.03	2,24 ± 0.11	2,10 ± 0.05	1,82 ± 0.47	1,74 ± 0.40
**K_{270}**	0,05 ± 0.02	0,05 ± 0.01	0,30 ± 0.04	0,21 ± 0.03	0,18 ± 0.14	0,13 ± 0.09
**ΔK**	0,00 ± 0.00	0,00 ± 0.00	0,00 ± 0.00	0,00 ± 0.00	0,00 ± 0.00	0,00 ± 0.00
**C16:0**	11,48 ± 0.09	11,40 ± 0.11	15,32 ± 1.40	15,44 ± 0.04	13,40 ± 2.37	13,42 ± 2.22
**C16:1**	0,79 ± 0.05	0,79 ± 0.09	1,35 ± 0.17	2,01 ± 0.02	1,07 ± 0.33	1,40 ± 0.67
**C17:0**	0,02 ± 0.00	0,04 ± 0.00	0,17 ± 0.09	0,06 ± 0.00	0,09 ± 0.11	0,05 ± 0.01
**C17:1**	0,07 ± 0.00	0,07 ± 0.00	0,19 ± 0.08	0,13 ± 0.00	0,13 ± 0.09	0,10 ± 0.03
**C18:0**	2,57 ± 0.14	2,89 ± 0.05	1,33 ± 0.29	0,78 ± 0.06	1,95 ± 0.72	1,84 ± 1.16
**C18:1**	78,26 ± 0.36	78,02 ± 0.36	69,48 ± 0.97	70,34 ± 0.09	73,87 ± 4.88	74,18 ± 4.22
**C18:2**	5,45 ± 0.22	5,42 ± 0.24	10,12 ± 0.51	9,63 ± 0.02	7,78 ± 2.59	7,52 ± 2.31
**C18:3**	0,45 ± 0.03	0,46 ± 0.04	1,04 ± 0.50	0,76 ± 0.00	0,74 ± 0.50	0,61 ± 0.17
**C20:0**	0,45 ± 0.02	0,45 ± 0.02	0,45 ± 0.01	0,36 ± 0.00	0,45 ± 0.02	0,41 ± 0.05
**C20:1**	0,25 ± 0.03	0,26 ± 0.02	0,32 ± 0.03	0,30 ± 0.01	0,29 ± 0.05	0,28 ± 0.03
**C22:0**	0,14 ± 0.00	0,14 ± 0.01	0,15 ± 0.01	0,12 ± 0.01	0,15 ± 0.01	0,13 ± 0.02
**C24:0**	0,05 ± 0.01	0,05 ± 0.01	0,09 ± 0.01	0.06 ± 0.00	0,07 ± 0.02	0,06 ± 0.01
**TChl (mg/kg)**	3,42 ± 0.61	2,70 ± 0.09	9,27 ± 1.96	9,69 ± 0.43	6,35 ± 3.58	6,19 ± 3.84
**TCa (mg/kg)**	2,53 ± 0.23	2,21 ± 0.12	7,42 ± 0.23	8,71 ± 0.48	4,98 ± 2.69	5,46 ± 3.58
**TP (mg/kg)**	986,49 ± 66.43	984,27 ± 38.79	569,86 ± 23.47	406.19 ± 6.82	778,18 ± 234.63	695,23 ± 318.09
**TT (mg/kg)**	548,00 ± 36.18	312,33 ± 8.38	641,47 ± 39.87	259.22 ± 15.93	594,74 ± 66.03	285,78 ± 32.26
**DPPH assay (% inhibition/mg)**	33,52 ± 4.70	27,74 ± 2.54	35,89 ± 1.30	24.41 ± 0.34	34.71 ± 3.65	26.07 ± 2.46
**ABTS assay (% inhibition/mg)**	57,60 ± 11.14	48,46 ± 7.33	33,75 ± 1.43	26.57 ± 0.47	45,67 ± 15.69	37.52 ± 13.27

Table 6. Qualitative characterization of the Ottobratica oils collected.

	**2017**		**2018**		**2019**	
**OTTOBRATICA**	**O**	**N**	**O**	**N**	**O**	**N**
**FA (oleic acid %)**	0,30 ± 0.03	0,46 ± 0.08	0,42 ± 0.01	0,47 ± 0.02	0,47 ± 0.08	2,62 ± 0.14
**PV (mEq O_2/kg)**	1,67 ± 0.24	1,88 ± 0.08	2,20 ± 0.14	7,53 ± 0.00	3,05 ± 0.18	10,47 ± 1.39
**K_{232}**	1,49 ± 0.04	1,37 ± 0.02	2,02 ± 0.14	2,53 ± 0.20	1,90 ± 0.06	1,64 ± 0.13
**K_{270}**	0,13 ± 0.00	0,11 ± 0.01	0,27 ± 0.02	0,24 ± 0.02	0,18 ± 0.01	0,24 ± 0.02
**ΔK**	0,00 ± 0.00	0,00 ± 0.00	0,00 ± 0.00	0,00 ± 0.00	0,00 ± 0.00	0,01 ± 0.00
**C16:0**	15,89 ± 0.17	14,33 ± 0.10	16,25 ± 0.07	15,11 ± 0.10	15,09 ± 0.72	15,44 ± 1.80
**C16:1**	1,37 ± 0.07	1,90 ± 0.03	1,45 ± 0.01	1,23 ± 0.00	1,93 ± 0.11	1,34 ± 0.44
**C17:0**	0,19 ± 0.01	0,02 ± 0.00	0,17 ± 0.00	0,16 ± 0.00	0,19 ± 0.23	0,12 ± 0.05
**C17:1**	0,31 ± 0.02	0,07 ± 0.01	0,30 ± 0.00	0,28 ± 0.01	1,56 ± 2.10	0,21 ± 0.09
**C18:0**	2,92 ± 0.12	2,06 ± 0.14	1,89 ± 0.32	1,25 ± 0.06	2,66 ± 1.27	2,00 ± 0.74
**C18:1**	67,59 ± 0.33	71,40 ± 0.59	68,51 ± 0.32	71,06 ± 0.13	69,39 ± 1.56	70,02 ± 4.78

**C18:2**	10,34 ± 0.14	8,83 ± 0.42	9,73 ± 0.04	9,15 ± 0.01	7,77 ± 0.35	9,43 ± 3.13
**C18:3**	0,52 ± 0.01	0,66 ± 0.03	0,69 ± 0.01	0,70 ± 0.00	0,64 ± 0.02	0,54 ± 0.02
**C20:0**	0,44 ± 0.00	0,35 ± 0.00	0,45 ± 0.01	0,46 ± 0.00	0,35 ± 0.02	0,44 ± 0.02
**C20:1**	0,22 ± 0.01	0,26 ± 0.02	0,31 ± 0.01	0,29 ± 0.00	0,26 ± 0.02	0,28 ± 0.02
**C22:0**	0,14 ± 0.00	0,10 ± 0.00	0,16 ± 0.00	0,15 ± 0.00	0,14 ± 0.02	0,16 ± 0.01
**C24:0**	0,06 ± 0.00	0,04 ± 0.00	0,10 ± 0.02	0,07 ± 0.00	0,02 ± 0.00	0,02 ± 0.00
**TChl (mg/kg)**	5,36 ± 0.52	2,13 ± 0.37	12,74 ± 1.93	3,90 ± 0.62	8,19 ± 2.27	4,52 ± 0.27
**TCa (mg/kg)**	4,03 ± 0.33	2,10 ± 0.37	6,97 ± 0.69	3,61 ± 0.49	6,16 ± 1.21	4,24 ± 0.06
**TP (mg/kg)**	345,06 ± 13.30	562,86 ± 108.44	188,15 ± 15.46	406,82 ± 1.39	1148,10 ± 127.89	32,90 ± 10.39
**TT (mg/kg)**	265,83 ± 40.50	237,99 ± 30.54	296,47 ± 16.70	264,20 ± 21.21	235,19 ± 33.52	211,78 ± 6.45
**DPPH assay (% inhibition/mg)**	28,42 ± 5.60	15,21 ± 1.53	17,08 ± 1.70	30,01 ± 0.05	20,42 ± 4.44	1,65 ± 0.40
**ABTS assay (% inhibition/mg)**	16,64 ± 1.42	59,85 ± 4.56	32,72 ± 0.97	29,78 ± 2.46	27,09 ± 3.40	6,57 ± 1.05
